# Autism Spectrum Disorder and Early Psychosis: a narrative review from a neurodevelopmental perspective

**DOI:** 10.3389/fpsyt.2024.1362511

**Published:** 2024-03-20

**Authors:** Silvia Guerrera, Maria Pontillo, Fabrizia Chieppa, Sara Passarini, Cristina Di Vincenzo, Laura Casula, Michelangelo Di Luzio, Giovanni Valeri, Stefano Vicari

**Affiliations:** ^1^ Child and Adolescent Neuropsychiatry Unit, Bambino Gesù Children’s Hospital, IRCCS, Rome, Italy; ^2^ Department of Dynamic and Clinical Psychology and Health Studies, Sapienza University of Rome, Rome, Italy; ^3^ Life Sciences and Public Health Department, Catholic University, Rome, Italy

**Keywords:** autism, schizophrenia spectrum disorder, neurodevelopmental continuum, positive symptoms, disorganization symptoms

## Abstract

Autism Spectrum Disorder (ASD), characterized by socio-communicative abnormalities and restricted, repetitive, and stereotyped behaviors, is part of Neurodevelopmental Disorders (NDDs), a diagnostic category distinctly in accordance with the Diagnostic and Statistical Manual of Mental Disorders, 5th Edition, (DSM-5), clearly separated from Schizophrenia Spectrum Disorder (SSD) (schizophrenia, schizophreniform disorder, schizoaffective disorder, schizotypal personality disorder). Over the last four decades, this clear distinction is gradually being replaced, describing ASD and SSD as two heterogeneous conditions but with neurodevelopmental origins and overlaps. Referring to the proposal of a neurodevelopmental continuum model, the current research’s aim is to provide an update of the knowledge to date on the course of clinical symptoms and their overlaps among ASD and SSD. A narrative review of the literature published between January 2010 and June 2023 was conducted. Five studies were included. All studies show a global impairment in both conditions. Two studies show a focus on neurodevelopmental perspective in ASD and SSD. Only one study of these adopts a longitudinal prospective in terms of prognostic markers among ASD and SSD. Three studies underline the overlap between ASD and SSD in terms of negative, disorganized and positive symptomatology. To date, there is a gap in the current scientific literature focused on ASD-SSD course of clinical symptoms and their overlaps from a neurodevelopmental perspective. Future longitudinal studies to identify risk markers and tailored treatments are needed.

## 1 Introduction

According to the Diagnostic and Statistical Manual of Mental Disorders, 5th Edition (DSM-5), Neurodevelopmental Disorders (NDDs) are defined as a group of condition whose onset is in the early developmental period. All NDDs are marked by developmental deficits leading to impairments in personal, social, academic, or occupational functioning. NDDs category includes intellectual disability (ID), Communication Disorders, Autism Spectrum Disorder (ASD), Attention-Deficit/Hyperactivity Disorder (ADHD), Neurodevelopmental Motor Disorders, and Specific Learning Disorders (1).

ASD is a NDDs characterized by persistent alterations in communication and social interaction and by the presence of stereotyped patterns of behaviors, activities, and interests (1). Besides difficulties in sociolinguistic communication and behavioral skills, the DSM-5 diagnostic criteria for ASD also encompass atypical sensory processing, characterized by either heightened or diminished reactivity to sensory stimuli. Schizophrenia, schizophreniform disorder, schizoaffective disorder, schizotypal personality disorder (SSD) are defined by symptoms such as delusions, hallucinations, disorganized or incoherent speech, grossly disorganized or abnormal motor behavior (including catatonia), and negative symptoms (1). Negative symptoms involve reduced intensity and range of emotional expression, diminished spontaneous speech and conversational fluency, decreased interest and motivation for productive activities, and a reduced desire for social interaction and affiliation. Additionally, negative symptoms encompass a reduced ability to experience pleasure and anticipate pleasure (2). More recently, negative symptoms have been classified into five constructs: blunt affect, alogia, anhedonia, asociality, and avolition (3). Of note, in individuals classified as Ultra-High-Risk (UHR) for psychosis, negative symptoms tend to appear before attenuated positive symptoms and their severity particularly can predict the likelihood of conversion to psychosis (4).

ASD and SSD show an historical connection. Eugene Bleuler (5) initially coined the term “autism” to describe the withdrawal from the external world observed in adult schizophrenia patients. Later, Kanner (6) employed the term to characterize a childhood behavioral disorder that he considered to be an early manifestation of schizophrenia. Rutter (7) claimed a clear distinction between ASD and SSD, a viewpoint that persisted for several decades. However, in the last four decades, this perspective has gradually evolved. Recent studies describe ASD and SSD such as two heterogeneous conditions with neurodevelopmental origins (8) exhibiting overlapping features. Nevertheless, they currently belong to two distinctly separate diagnostic categories. The reconceptualization of SSD as a psychopathological condition with origins before birth, displaying numerous prodromal and premorbid symptoms, and sharing neurobiological processes with other neurodevelopmental disorders (9) has led the current literature to focus on the overlaps between ASD and SSD. A recent study highlighted that Childhood-Onset Schizophrenia (COS) demonstrates premorbid disorders in the social and language domains in 67% of cases (10). Specifically, 27% of individuals with adult-onset schizophrenia met the criteria for ASD before onset, and some studies propose that the presence of these deficits may be considered a premorbid phenotype for COS (11).

Despite the distinct age of onset for ASD in childhood and SSD in early adulthood (1), research has consistently emphasized the convergence of ASD and SSD in terms of genetic and environmental factors (12). High heritability rates have been identified within each disorder (13–15) and between the two disorders. Notably, there is an elevated risk of ASD in children whose parents have a diagnosis of SSD (16, 17). Additionally, genetic studies on neuropsychiatric disorders have revealed an overlap in risk genes between schizophrenia (SCZ) and ASD (18). Ongoing epidemiologic investigations have identified an increasing number of risk genes and rare chromosome variants, such as microdeletions and duplications that are shared by both ASD and SSD (11). Moreover, ASD and SSD share several environmental risk factors, including complications during childbirth, gestational diabetes, low birth weight, congenital malformations, reduced head circumference, uterine atony, and asphyxia. Paternal age over 50 years has been identified as a potential risk factor for both ASD and SSD (19). Neuroimaging studies further support the connection between ASD and SSD, revealing shared deficits in brain regions involved in social cognition in both conditions. Specifically, a decrease in insula volume has been observed in both ASD and COS (20). Additionally, amygdala and medial prefrontal hypoactivation were noted in patients with both ASD and SSD (21).

In the past decade, genetic studies have provided evidence that NDDs share specific genetic risk alleles not only with each other but also with psychiatric disorders, particularly SCZ. This body of researches has given rise to the neurodevelopmental continuum model, suggesting that NDDs and adult psychiatric disorders can be conceptualized as existing on an etiological and neurodevelopmental perspective rather than being strictly defined as separate categories. The neurodevelopmental continuum model is grounded on the idea of shared genetic and environmental factors and overlapping pathogenic patterns. This model diverges from categorical diagnoses, which may fall short in capturing the nuanced boundaries of NDDs and their potential co-occurring conditions (22). Specifically, the neurodevelopmental gradient hypothesis classifies disorders according to the extent of neurodevelopmental impairment. This gradient is delineated by features such as age of onset, the severity of associated cognitive impairment, and the persistence of functional impairment, as outlined in the work by Owen et al. (23).

Despite the growing interest given to the relationship between ASD and SSD, the current literature has primarily concentrated on prevalence rates of co-occurrence and associations between positive symptoms and autistic traits. Few studies have delved into overlapping symptomatology, especially concerning also negative and disorganized symptoms. Bakken (24) has highlighted connections between SSD and ASD symptomatology, noting overlaps in incoherent, impoverished, and lacking in meaning speech, as well as disorganized speech in SSD potentially linked to ASD communication abnormalities. Shared behavioral aspects, such as violence, apraxia, and lack of meaningful gestures, have also been observed in both disorders (24). Furthermore, ASD abnormal preoccupation with stereotyped interests and motor mannerisms may parallel SSD delusions and disorganized/catatonic behavior (25).

Recent studies have paid attention to overlapping negative symptomatology between ASD and SSD, emphasizing that both disorders predominantly involve the absence of typical social and communicative behaviors (26, 27). Previously, Cochran and colleagues (25) also underlined that socio-communicative symptoms of ASD might be mistaken for the presence of psychotic symptoms. Features of ASD, such as impairments in nonverbal communication, lack of social reciprocity, and general social communication deficits, could be confused with social withdrawal, affective flattening, and negative symptoms characteristic of SSD. Poletti & Raballo (28) highlighted that despite the distinct ages of onset for ASD and SSD, there is a diagnostic grey area during development. Overlapping clinical symptomatology, including social distress and estrangement, may become evident, impacting not only early diagnosis but also the differentiation between childhood schizotypal traits (potential developmental precursors and risk factors for SSD) and high-functioning ASD. Moreover, reduced facial expression, body language, poor eye contact, and abnormal emotional expression are shared between ASD and Early Onset Schizophrenia (EOS) (27). This overlap is so significant that ASD patients often score high on the Positive and Negative Syndrome Scale (PANSS), while those with SSD exhibit high scores on the Autism Diagnostic Observation Schedule (ADOS-2) (27). Considering the literature reviewed and the recent introduction of a neurodevelopmental continuum model that focuses on the progression of clinical neurodevelopmental presentations towards psychiatric symptomatology, this narrative review aims to investigate the current understanding of the ASD-SSD course of clinical symptoms of the ASD-SSD and their overlaps, along with the clinical significance of negative, disorganized and positive symptomatology, from a neurodevelopmental perspective. The exploration of a potential connection between ASD and SSD holds paramount importance for diagnosis and treatment, as proposed by recent studies (29, 30). Specifically, recent literature has highlighted a positive correlation between the outcome and prognosis of individuals with SSD and the presence as well as the severity of premorbid neurodevelopmental difficulties. Recognizing individual clinical features beyond conventional categories has the potential to promote early diagnosis, deepen our understanding of psychological and pharmacological interventions, facilitate early and targeted interventions, and ultimately influence outcomes for individuals grappling with these disorders.

## 2 Methods

The current study consists of a narrative review of the literature published between January 2010 and June 2023.

### 2.1 Search strategy

An electronic database search via PubMed and Open Grey.EU was managed to find all included studies. Two different types of algorithms were used for the literature research: (autism) AND (schizophrenia); (autism) AND (psychosis).

5 articles were included. The last update of the search was in June 2023.

### 2.2 Inclusion and exclusion criteria

Included studies focus on overlapping symptomatology between ASD and SSD in samples of individuals aged 3-20. Inclusion criteria were: original research article, observational and experimental studies. We included only studies that referred to recognized diagnostic criteria (e.g., from Diagnostic and Statistical Manual of Mental Disorder four edition (DSM IV) to Diagnostic and Statistical Manual of Mental Disorder 5th edition text revision (DSM-5-TR) and in with clinical assessment for psychosis and autism based on psychometrically validated scale (e.g., Autism Diagnostic Observation Schedule-Generic (ADOS-G) or Schedule for Affective Disorders and Schizophrenia for school aged children present and lifetime version DSM-5(K-SADS-PL DSM5). Exclusion criteria were: article not in the area of interest (e.g., genetic focus or article for instrument validation) article format (reviews, meta-analyses, comments, and letters); sample characteristics (adult range age). Language restriction was applied (English).

### 2.3 Selection procedure, data extraction, and data management

**Figure 1:**
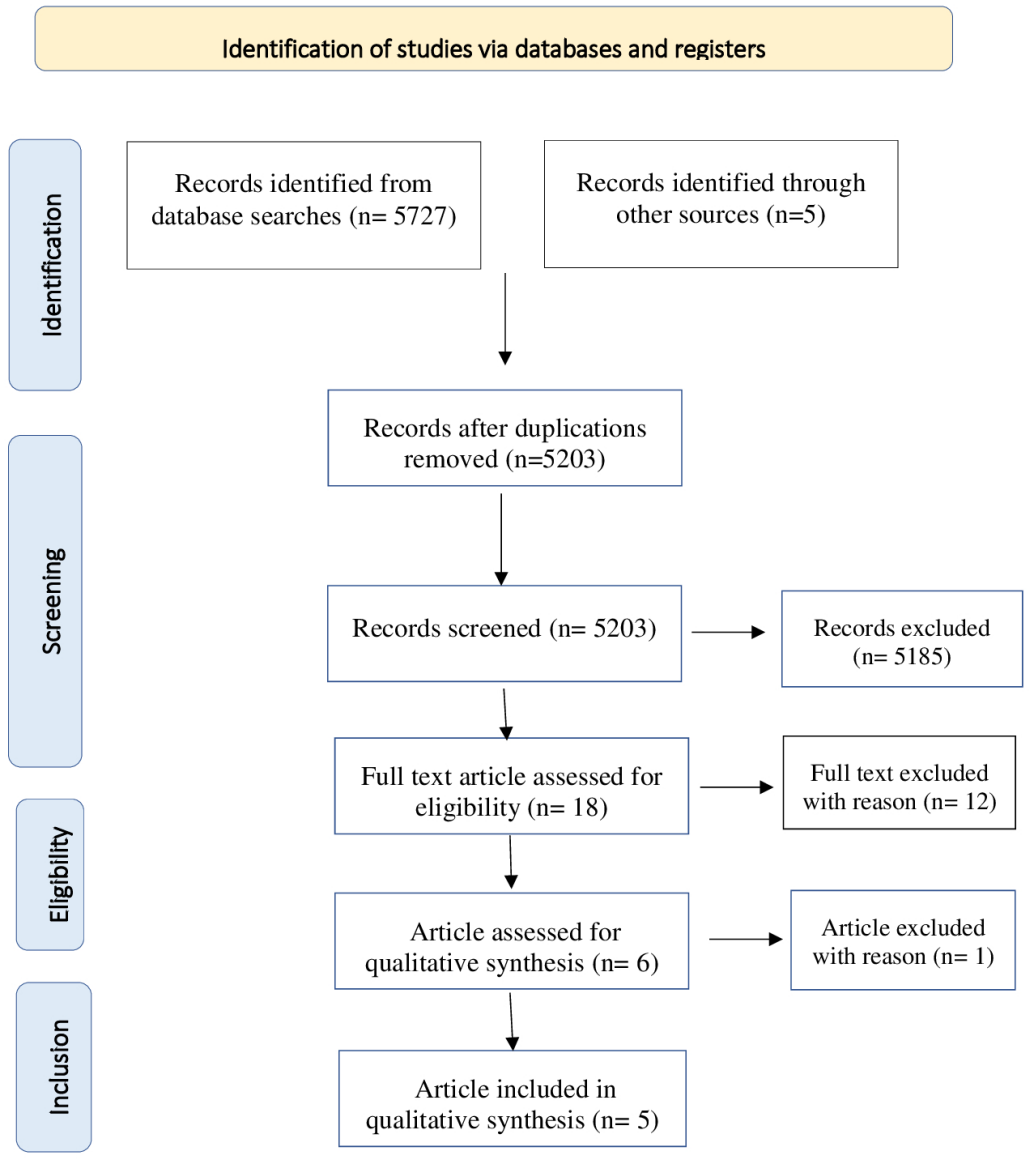
Flow chart of literature review using the same font for all its sections.

The reference lists of the most important articles of interest were examined. Studies were screened for eligibility by 2 authors independently (FC and SP). Possible eligible studies were identified on the basis of titles and abstracts. The potential target papers were assessed for eligibility on the basis of full text. Information on study design, sample size, inclusion and exclusion criteria, methods and results were extracted by FC and SP independently. Disagreements were sorted out in a consensus meeting with other reviewers (M.P and S.G). The search algorithm resulted a total of 5732 articles, of which 18 were examined as potentially eligible studies. 6 were retained for the final step, while the remaining 12 were excluded for the reasons listed (**Table 1**). Finally, 1 record was excluded due to sample’s not pediatric age. **Figure 1** presents a detailed flow diagram of the study selection process.

**Table 1:** Excluded studies and the reasons for their exclusion.

Reason of exclusion	Study Name
Article format (e.g., review)	(12) (31); (24) (26) (25) (9); (32); (33); (28); (34); (35);
Article not specific for age sample	(27)
Article not specific for clinical population sample	(36)

## 3 Results

We found a total of five studies on neurodevelopmental perspective and overlapping clinical features between ASD and SSD in children and adolescents, with a particular focus on negative, disorganized and positive symptomatology, for the period of time selected. Due to the low number of included studies, we proposed a narrative review.

Details on the methodology and results of the studies are shown respectively in **Figure 1** and **Table 2**.

**Table 2:** Methodologies and results of the investigated studies.

Study	Sample	Study Design	Inclusion Criteria	Measures	Results
Pontillo et al. (29)	N=176 Range age: 7-18 years	Cross sectional study	SSD according to DSM-5	K-SADS-PL SIPS/SOPS WISC CGAS GF-social scale GF-role scale	Neurodevelopmental disorder or difficulties showed an earlier onset of psychosis with a worst global functioning and strong correlations with SSD positive symptoms than individuals with VEOS/EOS and without any neurodevelopmental anomalies. Individuals without a neurodevelopmental disorder or difficulties showed a VEOS/EOS profile characterized by a solid negative symptomatology.
Bevan-Jones et al. (37)	N= 8232 (T0) N= 6439 (T1) Range age: 3-12 years.	Longitudinal study	ASD traits Early psychotic experiences	DAWBA; PLIKSi; WISC-III; The Bullying and Friendship Interview Schedule; Parental social class using 1991 OPCS classification; Family history of depression and schizophrenia.	Autistic traits at 3 and 7 years increase the likelihood of psychotic experiences at 12 years (p = 0.004). Speech problems, ritual, repetitive and restricted interests linked to ASD at the age of 3 and 7 increased the odds of psychosis traits at 12 years.
Barneveld (38)	N=27 ASD N= 30 TD Range age:10-18 years	Case control study	ASD according to DSM-IV-TR	AQ SPQ ADI-R WISC-III; WAIS-III	ASD group show significantly higher schizotypal traits schizotypal traits compared to controls (p<.001). ASD-SSD overlaps were mostly referred to negative schizotypal symptoms, but also extended to disorganized symptoms and positive symptoms.
Gadow (39)	N= 147 ASD N= 339 controls with ADHD or ODD Range age: 6-12 years.	Case Control Study	ASD according to DSM IV ADHD according to DSM IV SSD according to DSM IV	CASI-4R ADOS-G WISC	A moderate-strength correlation (0.30≤ rs ≤ 0.50) linked SSD negative symptomatology and ASD social and perseverative symptoms, intensified by ADHD co-occurrence. Similarly, SSD disorganized behavior showed a strong pattern of associations with ASD symptoms, strengthen by ADHD comorbidity. On the contrary, a weakest marked the association among SSD positive symptoms and the ASD symptomatology.
Solomon et al. (40)	N= 15 CHR N= 16 FEP N= 20 ASD N=20 TYP Range age: 11-20 years.	Cohort study	CHR according to DSM IV FEP according to DSM IV ASD according to DSM IV	WASI SIPS SCID-I/P ADOS-G SCQ CCC-2 SRS	All clinical groups (FEP, CHR, ASD) showed social reciprocity impairments compared to controls (ps<.01). FEP and ASD groups shared impairments in pragmatic and social relation subscales, differing significantly from both CHR (p =.01) and controls (p=.001). ASD group has the worst language profile compared to SSD and controls.

Schizophrenia Spectrum Disorder (SSD), The Diagnostic and Statistical Manual of Mental Disorders, Fifth Edition, (DSM-5), The Schedule for Affective Disorders and Schizophrenia for School-Age Children-Present and Lifetime version (K-SADS-PL), Wechsler Intelligence Scale for Children (WISC), Children’s Global Assessment Scale (CGAS), Global Functioning: Social Scale (GF-Social Scale), Global Functioning: Role Scale (GF-Role Scale), Autism Spectrum Disorder (ASD), the Development and Well-Being Assessment (DAWBA), Schizotypal Personality Questionnaire (SPQ), Psychosis-like symptoms semi-structured interview (PLIKSi), Wechsler Intelligence Scale for Children, Third Edition (WISC-III), The Diagnostic and Statistical Manual of Mental Disorders, Fourth Edition, Text Revision (DSM-IV-TR), Autism Spectrum Quotient (AQ), Sensory Perception Quotient (SPQ), Autism Diagnostic Interview-Revised (ADI-R), Wechsler Adult Intelligence Scale, Third Edition (WAIS-III), Attention Deficit Hyperactivity Disorder (ADHD), Child and Adolescent Symptom Inventory-4R (CASI 4-R), Autism Diagnostic Observation Schedule-Generic (ADOS-G), The Diagnostic and Statistical Manual of Mental Disorders, Fourth Edition (DSM-IV), Clinical High Risk for psychosis (CHR), First Episode Psychosis (FEP), Wechsler Abbreviated Scale of Intelligence (WASI), Structured Interview for Prodromal Symptoms (SIPS), The Structured Clinical Interview for DSM-IV Axis I Disorders (SCID-I/P), Social Communication Questionnaire (SCQ), Children’s Communication Checklist-2 (CCC-2), Social Responsiveness Scale (SRS).

### 3.1 Neurodevelopmental perspective in ASD and SSD

Pontillo et al. (29) investigated the relationship between neurodevelopmental disorders and early psychosis in a large sample (N=230) of children and adolescents (aged 7-18) admitted to Children Hospital Bambino Gesù in Rome. The sample included individuals with diagnosis of EOS, whose onset occurs between 13 and 17 years, or very early onset schizophrenia (VEOS), whose symptomatology onset occurs at of before 12 years.

The presence of psychiatric disorders was evaluated with the Schedule for Affective Disorders and Schizophrenia for school aged children present and lifetime version DSM-5(K-SADS-PL DSM5). Psychotic symptoms were assessed with the Structured Interview for Psychosis-Risk Syndrome (SIPS-SOPS). Finally the global functioning (family, school and social domains) through Childhood Global Assessment Scale (C-GAS).

Results showed that children and adolescents VEOS/EOS with neurodevelopmental disorder (e.g. autism spectrum disorder, intellectual disabilities, communication disorders) exhibited an earlier onset of psychosis (14.12 years). Additionally, this group showed a worst global functioning and higher scores on the positive symptoms scale of the SIPS/SOPS, specifically on the items grandiose ideas (p=.05), hallucinations (p=.025), and disorganized communication (p=.049) than children and adolescents VEOS/EOS without any neurodevelopmental disorders. Instead, children and adolescents VEOS/EOS without a neurodevelopmental disorder showed higher scores on the negative symptoms scale of the SIPS/SOPS, specifically on the items social anhedonia (p=0.001), expression of emotion (p=0.000), experience of emotion and self (p= 0.000) than children and adolescents VEOS/EOS with a neurodevelopmental disorders.

Finally, the most interesting result is that children and adolescents VEOS/EOS showed higher scores on the disorganized symptoms scale of the SIPS/SOPS, specifically on the items odd behavior or appearance (p= 0.016) and bizarre thinking (p= 0.017) than children and adolescents VEOS/EOS without a neurodevelopmental disorders. Bevan-Jones et al. (37) explored the association between subthreshold ASD symptoms and the onset of psychotic experiences during adolescence in 14541 children of the Avon Longitudinal Study of Parents and Children (ALSPAC) (41). Specifically, the study examined data from mothers who responded to questionnaires about their child at age 7.5 years and from 6455 children who attended the annual assessment at 12 years. Autistic traits were assessed using a parent interview named Development and Well Being Assessment (DAWBA). The onset of psychotic experience was evaluated through the psychosis-like symptoms semi-structured interview (PLIKSi).

Results underlined that among the 14541 cohort children, 8232 exhibited ASD traits. Of these 744 children (11.5%), over 6439 (78%) interviewed with the PLIKSi at 12 years, showed suspected or definite psychotic experiences. Autistic traits (speech and social problems, rituals) and the odd of psychotic experiences at 12 years are strongly associated (p = 0.004). Specifically, ASD traits referring to speech problems, ritual, repetitive and restricted interests at the age of 3 and 7 years increased the likelihood of psychosis traits at 12 years.

### 3.2 Overlap between ASD and SSD: the clinical significance of negative, disorganized and positive symptomatology

Barneveld et al. (38) investigated overlap between ASD and SSD traits in 27 ASD adolescents (aged 11-18) and in 30 typically developing (TD) controls, matched for age and gender. Of those with ASD, 11 were with schizotypal personality disorder co-occurrence. ASD traits were evaluated with Autism Questionnaire (AQ) while schizotypal traits with Schizotypal Personality Questionnaire- Revised (SPQ). Results showed in ASD group significantly higher schizotypal traits compared to controls (p<.001). Specifically, ASD-SSD overlaps were mostly referred to negative schizotypal symptoms (from 31% to 46%), but also extended to disorganized symptoms (from 13% to 19%) and positive symptoms (estimated 14%).

Gadow (39) investigated the associations between the severity of SSD, focusing SCZ and specific schizoid personality disorder, and ASD symptoms in terms of socio-communication deficits and perseverative behaviors. The search included a sample aged 6-12 of 147 children with ASD and 339 controls from child psychiatry outpatient, with or without Attention-Deficit/Hyperactivity Disorder (ADHD). Symptoms related to ASD and SSD were evaluated using the Child and Adolescent Symptom Inventory (CASI-4R), filled out by both parents and teachers. Ratings revealed a moderate-strength correlation between SSD negative symptoms (preference of being alone, little interest in relation and emotionally cold) and ASD social and perseverative symptoms (0.30 ≤ rs ≤ 0.50) intensify by ADHD co-occurrence. Also, SSD disorganized behavior (extremely odd things and behaviors) displayed a strong pattern of associations with ASD symptomatology, especially in case of ADHD comorbidity. Conversely, SSD positive symptoms such as hallucinations, delusions and disorganized thinking showed the weakest association with ASD symptomatology.

Solomon et al. (40) explored overlapping features among ASD and SSD focusing social-interaction impairments. Their sample consists in 71 subjects aged 11 to 20. Of these, 20 with ASD, 16 with a first episode of psychosis (FEP), 15 clinical high-risk for psychosis (CHR), and 20 TD controls. Diagnostic tools included the Structured Interview for Prodromal Symptoms (SIPS), the Structured Clinical Interview for DSM-IV Axis I Disorders (SCID-I/P), Autism Diagnostic Observation Schedule-Generic (ADOS-G), and the Social Communication Questionnaire (SCQ). Socio-interactional features were also assessed using the Social Responsiveness Scale (SRS) and the Children’s Communication Checklist (CCC-2). Results indicated social reciprocity impairments (in cognition, motivation, and mannerism) in all clinical groups compared to controls (ps<.01). Individuals with FEP and CHR resembled each other in social awareness and reciprocity communication, significantly differing from both controls (ps = .001) and ASD (ps = .05). In addition, FEP and ASD groups shared impairments in CCC-2 pragmatic and Social Relation subscales (friendship difficulties), differing significantly from both CHR (p =.01) and controls (p=.001). Nevertheless, subjects with ASD displayed much more language anomalies than SSD and controls, such as a failure to provide listener context and limited responsiveness.

## 4 Discussion

Implementing a neurodevelopmental perspective within an etiologic continuum, this narrative review focuses on overlapping symptomatology between NDDs, and specifically among ASD, and SSD during developmental age, focusing on clinical significance of negative, disorganized and positive symptomatology. Indeed, as previously highlighted in the literature (29, 30), exploring a potential connection between ASD and SSD can be of fundamental importance for diagnostic and therapeutic purposes. An approach that goes beyond categorical diagnostic boundaries and is able to capture premorbid symptoms could provide elements for an early diagnosis and suggest early and targeted interventions, promoting a better prognosis for these individuals. The five studies that we analyzed, indicate a global impairment both in the presence of SSD and ASD. Two studies focus on the neurodevelopmental perspective in ASD and SSD, revealing an early onset of SSD symptoms in patients with a neurodevelopmental disorder. The remaining studies highlight the overlap between ASD and SSD in terms of negative, disorganized, and positive symptomatology.

### 4.1 Neurodevelopmental perspective in ASD and SSD

Previous evidence by our research group (29) aligns with the neurodevelopmental continuum model and supporting its consistence with previous studies (10, 17). This body of works indicates that the presence of a neurodevelopmental disorder or difficulties, including motor, language, and social impairments, may be frequently associated with SSD symptomatology. Individuals with SSD and NDDs are characterized by an earlier onset and more severe functional impairment, encompassing social, role and school functioning. The aforesaid research, conducted on a large sample of children and adolescents with SSD, revealed that a significant proportion (37%) had a neurodevelopmental disorder. Analyzing the clinical profiles, it was observed that SSD patients with NDDs or difficulties, displayed more positive and disorganized symptoms compared to those without NDDs, who predominantly exhibited negative symptomatology. In particular positive symptomatology was characterized by grandiose ideas, perceptual abnormalities/hallucinations and disorganized communication. The disorganized symptomatology was mainly represented by odd behavior or appearance and bizarre thinking. In addition, children and adolescents with NDDs or difficulties exhibited impaired functioning in specific domains, such as social and role functioning. Consequently, the findings suggest that impaired social and communicative functioning is a common feature in children and adolescents who later develop SSD symptomatology. Furthermore, the aforementioned symptoms could serve as clinical markers for SSD patients preceded by NDDs or difficulties. In relation to this, future studies, preferably longitudinal, could replicate the results to enhance the understanding of clinical profiles and neurodevelopmental features in children and adolescents who develop SSD.

In a prior study, Bevan Jones and colleagues (37) explored the longitudinal progression of a large sample of children with ASD traits and tracking them into later adolescence to observe potential associations with SSD symptoms. Their findings revealed that the presence of ASD traits, particularly speech problems and rituals, at the age of 3, was linked to a higher probability of experiencing psychosis in adolescence. The persistence of these abnormalities at age 7 further strengthened this association. The authors presented three potential explanations for these observations: shared etiological mechanisms underlying ASD traits and psychotic experiences, the idea that ASD traits act as risk factors for the development of psychotic experiences, and the possibility that ASD and SSD represent facets of the same disorder manifesting differently at distinct developmental stages. Regardless of the chosen etiological interpretation, the authors proposed a neuroevolutionary continuum with overlapping symptomatology between ASD and SSD. This challenges current diagnostic systems and underscores the need for increased awareness regarding the connections between various NDDs and adult psychiatric disorders, explaining the diverse presentations seen during developmental age. In summary, this narrative review, consistent with prior evaluations, brings to light symptomatic features such as speech difficulties, rituals, and repetitive and restricted interests. These traits appear to be linked to the potential development of psychosis later on. Future studies could delve into these data and assess the presence of risk markers that may indicate clinical course of symptoms and their overlaps with SSD. Confirmation could support early intervention focused on addressing the impairment in these areas of development.

### 4.2 Overlap between ASD and SSD: the clinical significance of negative, disorganized and positive symptomatology

Studies conducted by Barnevald (38), Salomon (40), and Gadow (39), along with their colleagues, explored the overlap of clinical features between ASD and SSD in children and adolescents, particularly focusing on impairments in specific domains and in reference to negative, disorganized, and positive symptoms. Barnevald and colleagues (38) discovered significantly higher traits of SSD in the ASD group, particularly in terms of positive, negative (including constricted affect and social anxiety), and disorganized symptoms compared to controls with typical development. Of note, ASD traits related to ASD domains, such as poor social skills, attention switching, communication, and imagination, were more strongly correlated with negative symptoms of SSD than with positive and disorganized symptoms.

Other studies, including those by Gadow (39), Solomon (40), as well as the one previously discussed of Pontillo et al. (29), provide valuable insights into the interconnected relationship between ASD and SSD during developmental age, emphasizing the complexity and potential continuum between these neurodevelopmental disorders. Gadow’s study (39) revealed a significant correlation between schizoid personality disorder symptoms within SSD and social deficits observed in ASD. Additionally, investigations into SSD in terms of negative, positive and disorganized symptoms of SCZ demonstrated that negative and disorganized symptoms were more strongly related to the social deficits of ASD than positive symptoms. Solomon and colleagues (40), aiming to recognize early impairments in social and language functioning in children and adolescents who later developed FEP and CHR, placed symptoms of FEP and CHR conditions between ASD and healthy controls in a fine gradient for social impairments in terms of cognition, motivation, and mannerism. The ASD group displayed the most pronounced deficits in social awareness and communication, while both FEP and ASD groups demonstrated comparable impairments in pragmatic language and social relationships—interpreted as challenges in forming friendships, when compared to both TD controls and individuals with CHR. Finally patients with FEP and CHR exhibited similarities in social awareness and reciprocal communication, demonstrating significant differences from both TD controls and ASD. Authors concluded that individuals with ASD, CHR, and FEP shared common features of atypical neurodevelopment, particularly significant impairments in communication and social function.

The results of Salomon and colleagues seem to indicate that social impairment can be considered a cross-cutting symptom between SSD and ASD as it emerges in the neurodevelopmental perspective from ASD to SSD. The subsequent symptomatic characterization is enriched with positive and disorganized symptoms. To support this data, Sullivan et al. (17) clarified that both psychotic experiences and autism exhibit impairment in social cognition, which, in turn, plays a mediating role in the development of social interaction skills. The same overlap in impairment between the two conditions has been demonstrated for language pragmatics. In particular, the authors report a theory of mind difficulty observed in individuals with both ASD and SSD, crucial for the acquisition of communicative skills. Concluding, of five studies included in our revision, all show a global impairment in both conditions, ASD and SSD. Two studies show a focus on neurodevelopmental perspective in ASD and SSD that goes beyond discrete clinical entities. This would support the understanding of complex clinical pictures with nuanced or ill-defined symptoms, exhibiting different types of onset and responses to interventions. One of these two studies adopts a longitudinal prospective in terms of prognostic markers among ASD and SSD focusing the attention on possible explanations for the association observed: that common risk factors, increased vulnerability conditions, or manifestations of the same disorder at different developmental ages are proposed explanations. Three studies focus on impairments in specific domains and the overlap ASD SSD in terms of negative, disorganized and positive symptoms, evidencing that a detailed understanding of which could provide tools for early diagnosis and specific treatment. However, it’s crucial to acknowledge several limitations in this review. First of all, our search revealed a very limited number of recent studies investigating overlapping symptomatology between ASD-SSD with refers to the symptomatic characterization during developmental age, which may be bias the results. Secondly, the heterogeneity observed across studies in terms of objectives, clinical features classified within the SSD or ASD category, or conditions defined as ASD traits. Thirdly, the use of different measures for clinical assessment posing a challenge in synthesizing the results. These do not allow a quantitative approach for data extraction and analysis and limit the interpretability of the findings. Moreover, the cross-sectional nature of most studies limits the ability to draw prognostic implications from a neurodevelopmental perspective. Despite these limitations, the results of this narrative review suggest that clinicians should investigate potential SSD symptomatology in individuals with ASD diagnosis. On the flip side, clinicians should also investigate NDDs, ASD, in individuals exhibiting symptoms of SSD. The emerging evidence, which indicates a link between NDDs and adult psychiatric conditions, emphasizes the significance of systematic screening assessments. This information might not be currently considered in clinical routines, referencing a categorical diagnostic classification that deems psychotic disorders as rare during childhood (even though psychotic experiences are not) (1). Therefore, in our opinion, while recognizing the need to observe diagnostic categories in psychopathology based on a symptomatic reading that essentially reflects important guidelines for the clinician’s work, utilizing an approach that considers an interpretation of clinical symptoms within a neurodevelopmental continuum model in children and adolescents could facilitate a more detailed understanding of the clinical profile of these individuals. It would also be useful to consider that the same DSM, in the introductory part of its fifth edition (1), compared to previous editions, emphasizes the need for continuity between developmental psychopathology and that of adults highlighting the concept that a disorder present in development cannot fail to leave a trace in adulthood (42). Considering neurodevelopmental continuum model would also allow for the identification of premorbid symptoms at onset, enabling the development of more specific pharmacological and psychological interventions, ultimately altering outcomes for individuals with ASD-SSD in terms of the clinical course of symptoms and their overlaps. Finally, considering the gap of literature, future longitudinal studies to identify risk markers and promote treatment individualization.

## Author contributions

SG: Conceptualization, Writing – original draft, Writing – review & editing. MP: Conceptualization, Writing – original draft, Writing – review & editing. FC: Data curation, Methodology, Writing – review & editing, Investigation. SP: Data curation, Methodology, Writing – review & editing, Investigation. CDV: Data curation, Methodology, Writing – review & editing. LC: Data curation, Writing – review & editing. ML: Data curation, Writing – review & editing. GV: Conceptualization, Writing – review & editing. SV: Conceptualization, Writing – review & editing.
